# Illustrated formalisms for total scattering data: a guide for new practitioners

**DOI:** 10.1107/S1600576720015630

**Published:** 2021-02-01

**Authors:** Peter F. Peterson, Daniel Olds, Marshall T. McDonnell, Katharine Page

**Affiliations:** a Computer Science and Mathematics Division, Oak Ridge National Laboratory, Oak Ridge, TN, USA; b National Synchrotron Light Source-II, Brookhaven National Laboratory, Upton, NY, USA; c Neutron Scattering Division, Oak Ridge National Laboratory, Oak Ridge, TN, USA; d Materials Science and Engineering Department, University of Tennessee, Knoxville, TN, USA

**Keywords:** total scattering, pair distribution function

## Abstract

The total scattering method is the simultaneous study of both the real- and reciprocal-space representations of diffraction data. While conventional Bragg-scattering analysis (employing methods such as Rietveld refinement) provides insight into the average structure of the material, pair distribution function (PDF) analysis allows for a more focused study of the local atomic arrangement of a material. Generically speaking, a PDF is generated by Fourier transforming the total measured reciprocal-space diffraction data (Bragg and diffuse) into a real-space representation. However, the details of the transformation employed and, by consequence, the resultant appearance and weighting of the real-space representation of the system can vary between different research communities. As the worldwide total scattering community continues to grow, these subtle differences in nomenclature and data representation have led to conflicting and confusing descriptions of how the PDF is defined and calculated. This paper provides a consistent derivation of many of these different forms of the PDF and the transformations required to bridge between them. Some general considerations and advice for total scattering practitioners in selecting and defining the appropriate choice of PDF in their own research are presented. This contribution aims to benefit people starting in the field or trying to compare their results with those of other researchers.

## Abstract 2

This article provides a detailed and visual presentation of the derivations of and relationships between many of the commonly employed functional forms of real- and reciprocal-space data employed by the worldwide total scattering community.

## 1. Introduction   

Pair distribution function (PDF) analysis is a broad term encompassing the use of experimentally generated atomic pair density functions in real space to study short-range order in materials. The approach has its roots in the work of Warren and co-workers, who developed it to study the short- and intermediate-range structure of glasses (Warren, 1934 ▸, 1937 ▸). Notable extensions have included work on molecular liquids (Narten, 1972 ▸), the study of glasses and crystalline materials (Srolovitz *et al.*, 1981 ▸), the study of liquids and glasses with neutron diffraction (Wright *et al.*, 1989 ▸; Wright, 1990 ▸, 1994 ▸), the use of high-energy X-rays (Egelstaff, 1967 ▸; Root *et al.*, 1986 ▸; Neuefeind & Poulsen, 1995 ▸; Neuefeind *et al.*, 1996 ▸), the adoption of the technique for reverse Monte Carlo (RMC) methods (McGreevy & Pusztai, 1988 ▸; McGreevy, 1995 ▸, 2001 ▸), and the application to nanostructured and disordered crystalline materials (Billinge, 1992 ▸; Egami & Billinge, 2012 ▸). These advancements and their applications to materials are distinguished in a number of review articles and text books appearing in the past two decades, for liquids and glasses (Neuefeind, 2002 ▸; Egelstaff, 2003 ▸; Kohara & Suzuya, 2003 ▸; Fischer *et al.*, 2005 ▸; Kohara *et al.*, 2007 ▸; Benmore, 2012 ▸), and for disordered crystalline and nanostructured materials (Egami, 2007 ▸; Billinge & Kanatzidis, 2004 ▸; Billinge, 2004 ▸; Billinge & Levin, 2007 ▸; Young & Goodwin, 2011 ▸; Egami & Billinge, 2012 ▸; Playford *et al.*, 2014 ▸; Hou *et al.*, 2018 ▸).

The PDF is a probability distribution function that measures the probability of finding pairs of atoms separated by a given distance. There are many different forms of the PDF with subtly different functional forms, units, normalizations and use in research communities, but they all contain the same information: the probability of finding atoms separated by a distance (Dinnebier & Billinge, 2008 ▸). For example, from a statistical mechanics definition of the radial distribution function, this probability can be given relative to the ideal gas state, where no correlations exist. Therefore, deviations in such a distribution function will give a factor to multiply the bulk density by to get a local density (McQuarrie, 2000 ▸). All the different forms of the PDF give information about the changes in local density with distance and thus insight into the local structure.

The use of PDF analysis has grown exponentially from a specialized technique employed for the study of liquids, glasses and other amorphous materials [where Rietveld (1969 ▸) analysis is not possible] to one that encompasses disordered materials more broadly through the study of local atomic structure and disorder in nanocrystalline and crystalline materials. As various material communities have adopted PDF analysis, they have refined the methodology and developed corresponding analysis software to address their specific scientific needs. The weighting and normalization of different features, either from the measured reciprocal-space data or modeled real-space atomic coordinates, has led to no less than eight different published forms of real-space distribution function (Keen, 2001 ▸), which can all claim in one way or another to be analogous to the PDF. Despite being functionally similar, the differences between these varied forms has led to some confusion and redundancy across the different communities.

This contribution aims to untangle many of these semantic and terminological confusions through a consistent derivation of the relationships between many different forms of the PDF, demonstrated through simple examples. We reintroduce many fundamental concepts and formalisms, and directly relate them to the physical and distinguishing features they represent.

The equations are presented here with neutron scattering formalism, where there is no *Q* dependence in the atomic scattering lengths. The added complexity brought by *Q*-dependent X-ray form factors (Narten, 1972 ▸) has been, broadly, addressed in three approaches: explicit corrections during data normalization (Qiu, Thompson & Billinge, 2004 ▸), *ad hoc* or approximative corrections (Juhás *et al.*, 2013 ▸; Billinge & Farrow, 2013 ▸) during data normalization, or leaving the data uncorrected and instead forward calculating the effects in a refined model (Gereben *et al.*, 2007 ▸; Tucker *et al.*, 2007 ▸). The variability of these different approaches and their implications are outside the scope of this paper. Herein, the presented derivations assume that the reduction of X-ray scattering data successfully mitigates the effects of these form factors.

For the remainder of this text, the authors make the following assumptions which are being made explicit with the aim of aiding the reader even further in seeing the bridge between various conventions. First, the term ‘total scattering’ was adopted for the PDF method within the past few decades to bring attention to the fact that it provides an examination of both Bragg and diffuse scattering. The modern adoption of this colloquial name should be distinguished from the foundational terminology ‘total scattering’ in use by the general time-of-flight neutron scattering community (Squires, 2012 ▸). Note also that most neutron and X-ray PDF measurements are energy-integrated scattering functions. Throughout this manuscript ‘total scattering’ will be used to refer to the PDF technique in general. Second, as will be further explained in Section 3, the *I*(*Q*) presented here is proportional to the differential cross section. Note that *I*(*Q*) is different from the measured intensity often employed in Rietveld refinements, sometimes utilizing the same nomenclature (Rietveld, 1969 ▸).

Previous work went into great detail on the derivation and conversion of different functional forms of the PDF, and is widely cited in the community (Keen, 2001 ▸; Fischer *et al.*, 2005 ▸). Subtle differences in nomenclature of various approaches have led to some confusion in the PDF communities, particularly when converting between the different formalisms. This manuscript expands on this work, beginning with the conventions of the disordered crystalline material communities (Egami & Billinge, 2012 ▸) and bridging to other derivations. Visual comparisons of various functional forms guide the reader and frame discussion of each use case. Effort has been made to reference both initial derivations and examples of utilization in scientific literature.

The derivations and examples in this paper are presented first in reciprocal space then in real space. A collection of appendices provide both reference and further detail supporting the derivations. Appendix *A* [App appa] provides a list of simple conversions between the various real-space functions. Appendix *B* [App appb] provides details of the molecular dynamics simulations used for the liquid example data. Appendix *C* [App appc] is an overview of the process of converting from measured intensities to differential cross sections to provide a frame of reference for understanding experimental concerns. Appendix *D* [App appd] details calculation of the normalized Laue term employed in some formalisms, while Appendix *E* [App appe] details calculation of the number density. Appendix *F* [App appf] provides a brief overview of partial structure functions.

## 2. Methods   

To illustrate the different functions contained herein, we have simulated neutron nuclear scattering data for two systems of representative material types: bulk binary oxide manganese oxide (MnO) (Sasaki *et al.*, 1979 ▸) for crystalline and disordered materials; and liquid argon (Ar) (Yarnell *et al.*, 1973 ▸) for liquid, amorphous and glass materials. Mn has a negative neutron scattering length and O has a positive one, which emphasizes certain differentiating characteristics of various forms of the PDF. MnO has a magnetic structure that will be ignored to allow for focusing on the atomic structure. Simulated data from liquid argon (Ar) are also included as a monatomic example. Note that the term ‘monatomic system’ here assumes a single element with a single isotope for neutron scattering and a single element with a single charge state for X-ray scattering.

To generate the presented MnO data, real-space patterns were simulated with the *PDFgui* software (Farrow *et al.*, 2007 ▸) using the crystal structure in Table 1 ▸. Patterns were calculated for 0 ≤ *r* ≤ 160 Å with a bin width of δ*r* = 0.01 Å. This pattern was then inverse transformed to generate the presented reciprocal-space data.

To generate the Ar data, molecular dynamics simulations were performed using the *Large-Scale Atomistic/Molecular Massively Parallel Simulator* (*LAMMPS*) open-source code (Plimpton, 1995 ▸, 2018 ▸). Details of the simulations are provided in Appendix *B* [App appb].

## 3. Reciprocal-space functions   

We begin the derivation assuming data in the form of the fully corrected and normalized scattering intensity, *I*(*Q*), obtained from experimentally measured intensities. Interestingly, Debye himself, along with Menke, performed the first PDF experiments using X-ray scattering in 1930 to obtain such data (Debye, 1930 ▸). The details for a protocol used to reduce such measured data to fully corrected patterns based on literature and reduction software manuals can be found in Appendix *C* [App appc]. *I*(*Q*) can be directly related to a set of atomic coordinates through the Debye (1915 ▸) scattering equation: 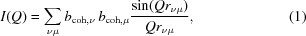
where *b*_coh,ν_ is the coherent scattering length of atom ν and *r*_νμ_ = |**r**_ν_ − **r**_μ_| is the interatomic pairwise vector of atoms ν and μ (Lovesey, 1986 ▸; Farrow & Billinge, 2009 ▸; Page *et al.*, 2011 ▸). Debye’s formalism, slightly modified to include the effects of thermal atomic displacements through a Debye–Waller term σ_νμ_ (Debye, 1913 ▸; Waller, 1923 ▸), is written as 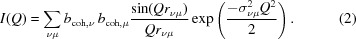
In this formalism *I*(*Q*) is the scattering from the sample as a whole. The total scattering community most commonly employs *I*(*Q*)/*N* (the differential cross section), where *N* is the number of atoms illuminated in the sample (described further in Appendix *E* [App appe]).

Peak profile refinement methods (*e.g.* Rietveld analysis) most commonly define the scattering per sample, rather than scattering per atom. This convention can be traced back to Rietveld’s initial aims of fitting models against the peak profiles of the relative intensities as directly generated from instrument measurements. Use of an arbitrary scale factor during modeling was convenient and sufficient for this purpose. The total scattering formalism, on the other hand, allows for a fully atomistic model comparison with data. Accurate corrections to remove experiment artifacts are required to compare the data with atomistic models, the importance of which has been shown elsewhere (Egelstaff, 1992 ▸; Wright, 1994 ▸; Fischer *et al.*, 2005 ▸). However, in practice, many practitioners studying crystalline materials also apply an arbitrary scale factor to data during modeling (Farrow *et al.*, 2016 ▸). In fact, the departure of a scale factor from unity for standard (known) samples is sometimes applied as a quality criterion for assessing the success of data reduction procedures (Peterson *et al.*, 2003 ▸).

When defined only through isotropic atomic displacement parameters (commonly referred to as *U*_iso_), the Debye–Waller term σ_νμ_ can be written as (Jeong *et al.*, 1999 ▸, 2003 ▸; Proffen & Billinge, 1999 ▸) 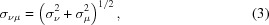
where σ_ν_ and σ_μ_ are the amplitudes of the uncorrelated thermal motion of atoms ν and μ. This relationship is more complicated in the case of anisotropic atomic displacement (Dunitz *et al.*, 1988 ▸; Jeong *et al.*, 2003 ▸), but its effect on the normalized intensity is similar: exponential dampening of the Bragg intensities at high *Q*.

Another form of the normalized and corrected scattering data is the ‘structure function’, *S*(*Q*). This form of the scattering data is employed in the generation of many atomic pair–pair representations of data, which accounts for its widespread description in past work (Yarnell *et al.*, 1973 ▸; Billinge & Egami, 1993 ▸; Keen, 2001 ▸; Peterson *et al.*, 2003 ▸; Farrow & Billinge, 2009 ▸; Page *et al.*, 2011 ▸; Olds *et al.*, 2015 ▸) where details of the derivation can be found. The structure function is related to the normalized *I*(*Q*) function through the relationship 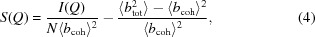
where 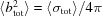 is the average total scattering power of the system, σ_tot_ is the total cross section and 〈*b*_coh_〉^2^ is the average coherent scattering power of all atoms in the sample. Appendix *D* [App appd] provides a more complete discussion of the total scattering length term, 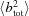. The second term in equation (4) contributes a constant factor called the normalized Laue monatomic diffuse scattering term. The normalized Laue term, often written simply as *L*, is 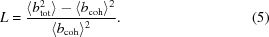

*L* is zero in the case of single-element scattering (though not strictly so for neutron scattering, since naturally abundant elemental samples often comprise a mix of isotopes). *L* is calculated for our simulated examples in Appendix *D* [App appd].

The limits of *S*(*Q*) are 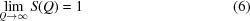
and 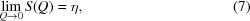
where η is unitless and proportional to the isothermal compressibility of the sample (Lovesey, 1986 ▸; Egelstaff, 1992 ▸; Wang *et al.*, 2014 ▸). Thermodynamically, η is defined as 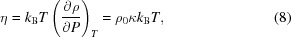
where κ is the isothermal compressibility (equal to the inverse of the bulk modulus, *K*_0_), ρ_0_ is the number density, *k*_B_ is Boltzman’s constant, *T* is temperature and *P* is pressure. This is only strictly correct for monatomic, homogeneous, isotropic systems and is incorrect for a fluid close to its critical point. Additional details and references for more complex cases such as mixtures of molecular liquids and ions in aqueous solution are given by Fischer *et al.* (2005 ▸). The isothermal compressibility can be calculated as 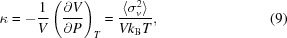
where *V* is the volume and 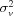 is the variance of the volume. η is often negligible (Bhatia & Thornton, 1970 ▸; Wagner, 1985 ▸; Egelstaff, 1992 ▸; Keen, 2001 ▸), as shown in Table 2 ▸ for our simulated examples. Note that in cases where nanostructured features exist (such as materials where small-angle scattering is present) the measured low-*Q* behavior will deviate (Mildner & Carpenter, 1984 ▸; Farrow & Billinge, 2009 ▸; Olds *et al.*, 2015 ▸).

The ‘reduced total scattering structure function’ is defined as 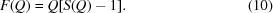
This representation of reciprocal-space data has a limit of 0 at high *Q* and is linearly weighted by *Q*, such that noise and resolution are highlighted features that have dramatic effects on the resultant real-space PDF). Another advantage to this formalism is that associated uncertainties increase linearly with *Q* (Egami & Billinge, 2012 ▸; Olds *et al.*, 2018 ▸).

**Figure 1:**
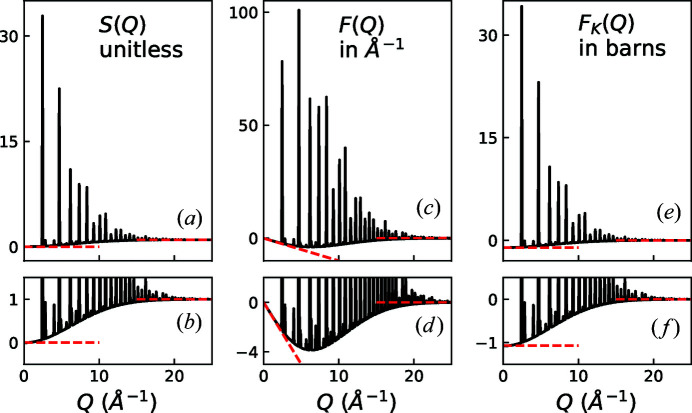
Comparison of reciprocal-space representations of MnO total scattering data: (*a*), (*b*) *S*(*Q*), (*c*), (*d*) *F*(*Q*) = *Q*[*S*(*Q*) − 1], and (*e*), (*f*) *F*_K_(*Q*) = 〈*b*_coh_〉^2^[*S*(*Q*) − 1]. The upper plots show an overview of the various functions. The asymptotes are highlighted with dashed lines. In this specific case, 〈*b*_coh_〉^2^ = 1.074 fm^2^ such that the difference between *S*(*Q*) and *F*_K_(*Q*) coincidentally appears to be a vertical shift of one (1). Also note that for MnO η = 0.0013, which appears to be zero on the scale of this figure.

**Figure 2:**
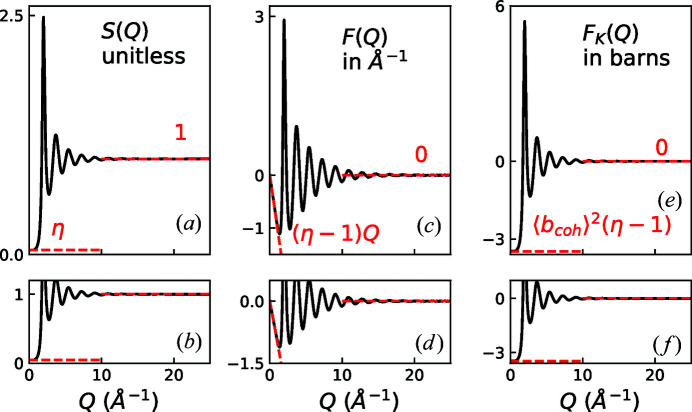
Comparison of reciprocal-space representations of Ar total scattering data: (*a*), (*b*) *S*(*Q*), (*c*), (*d*) *F*(*Q*) = *Q*[*S*(*Q*) − 1], and (*e*), (*f*) *F*_K_(*Q*) = 〈*b*_coh_〉^2^[*S*(*Q*) − 1]. The upper plots show an overview of the various functions. The asymptotes are highlighted with dashed lines.

In the derivations of Keen (2001 ▸), a similar function also referred to as *F*(*Q*) is presented. This alternative function, here referred to as *F*_K_(*Q*), is scaled by 〈*b*_coh_〉^2^ and not by *Q*. Thus, these three reciprocal-space function are related as 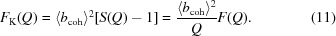
The normalized and corrected intensity, *I*(*Q*), is related to *F*(*Q*) and *F*_K_(*Q*) as 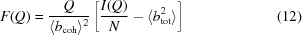
and 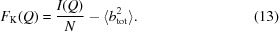
A visual comparison of *S*(*Q*), *F*(*Q*) and *F*_K_(*Q*) is shown for the case of MnO in Fig. 1 ▸ and for the case of Ar in Fig. 2 ▸. A summary of the limiting behaviors of these functions and *I*(*Q*) can be found in Table 3 ▸.

## 4. Real-space distribution functions   

The pair distribution function is a general concept describing the distribution of distances between pairs of objects contained in a volume. Zernike & Prins (1927 ▸) were the first to report the theoretical expression for the atomic density at a given separation in real space via their Fourier transform relationship, leading to the origin of the PDF formalisms. Yet, throughout the years of literature on the PDF, many different functional forms have spawned from this origin.

**Figure 3:**
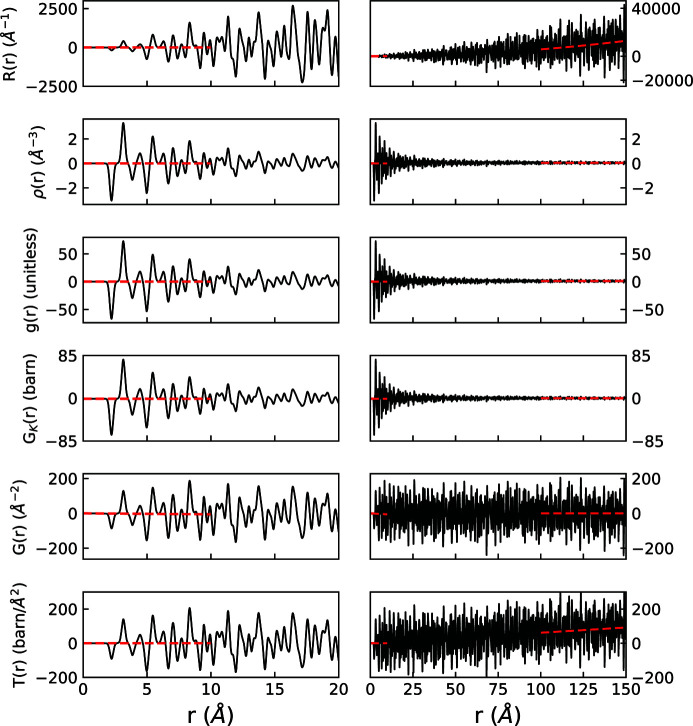
Comparison of long-range behavior of different real-space functions for MnO. The dashed lines highlight the asymptotic behavior. Since *D*(*r*) = 〈*b*_coh_〉^2^ *G*(*r*), it is not shown.

**Figure 4:**
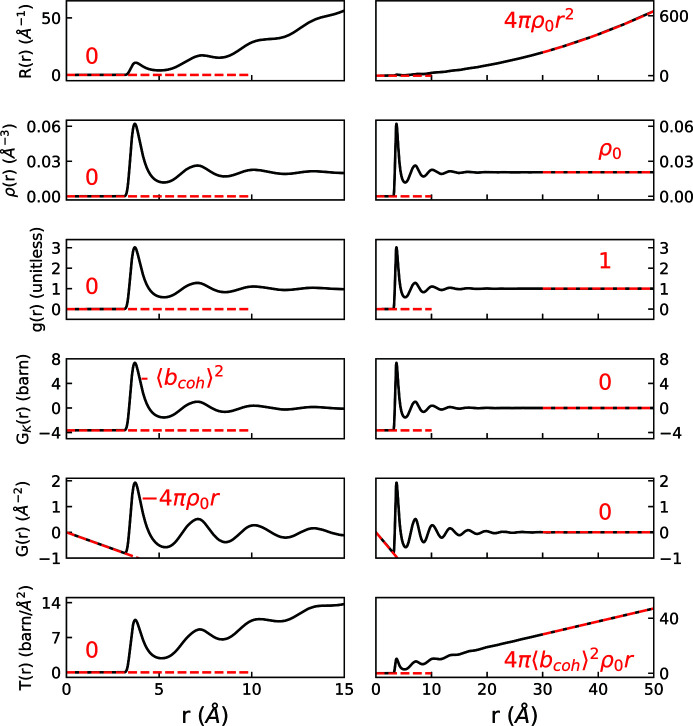
Comparison of long-range behavior of different real-space functions for Ar. The dashed lines highlight the asymptotic behavior. Since *D*(*r*) = 〈*b*_coh_〉^2^ *G*(*r*), it is not shown.

When defined independently of the atomic origin, μ, this is termed a radial distribution function (RDF), an entity that finds prevalent use as a descriptor for the atomic structure of amorphous, liquid, disordered and nanocrystalline materials. The same name can be associated with different functional forms, ever increasing confusion. For example, the name ‘radial distribution function’ is associated with both equation (14) and equation (22) in previous literature (Thorpe *et al.*, 1998 ▸; McQuarrie, 2000 ▸). In this section, we derive and relate a number of functions used in various research communities for representing real-space PDFs (see Figs. 3 and 4 below). These are generally related by multiplicative or additive constants and thus contain the same underlying information. We will explain some of the relative merits and related preferences for these formalisms at the close of the section.

A conceptually different quantity is the RDF which, containing no relationship to scattering weights and thus not directly measurable, is presented here for comparison. We begin by defining a configuration of *N* atoms arranged such that each atom has a position defined through the vector **r**_ν_. The interatomic distance between any pair of atoms, μ and ν, is thus *r*_μν_ = |**r**_ν_ − **r**_μ_|. An unweighted radial distribution function, labeled here RDF(*r*), can be constructed through the sum of Dirac delta functions, δ, which describe the full set of these pair–pair distances [of which there will be *N*(*N* − 1)/2 total pairs]. RDF(*r*) can be written as 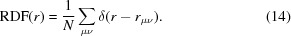
A radial PDF can be generated from the measured scattering intensities of various physical measurements, including light scattering, electron diffraction, X-ray diffraction and neutron diffraction, with the last three all used to produce atom–atom PDFs. RDF(*r*) is straightforward to calculate but is only straightforward to measure with monatomic systems. More easily measured, radiation-specific PDFs can be calculated from atomic models by accounting for the scattering power of each atom. This results in the weighted radial distribution function, *R*(*r*), defined as 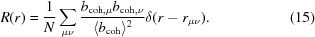
For the case of monatomic systems, the weighting prefactor becomes unity and *R*(*r*) simplifies to the equation for RDF(*r*).

A similar formalism often encountered is the density function, ρ(*r*), which is the radial distribution function normalized by the surface area of a sphere of radius *r*, such that (Warren, 1990 ▸) 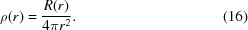
For isotropic and three-dimensional systems, the density function can be directly related to *S*(*Q*) through the following pair of transforms (Warren, 1990 ▸; Billinge, 1992 ▸): 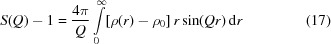
and 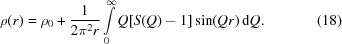
The heavily used form of the PDF encountered in disordered crystalline material literature is the reduced pair distribution function, *G*(*r*) (Egami & Billinge, 2012 ▸), which is defined in relation to the density function as 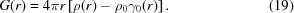
Here, ρ_0_ is the average number density of *N* atoms in the volume *V* such that ρ_0_ = *N*/*V*, and γ_0_(*r*) is the characteristic shape function or nanoparticle form factor (Guinier & Fournet, 1955 ▸; Azaroff, 1968 ▸; Farrow & Billinge, 2009 ▸; Olds *et al.*, 2015 ▸). In the case of bulk materials, γ(*r*) = 1.0, and thus the term is often neglected in the literature.

This reduced pair distribution function can be generated from reciprocal-space data via the sine transform of *F*(*Q*), such that 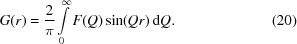
Therefore, the Fourier inversion theorem holds that 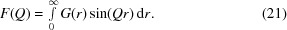



An alternative formalism of the PDF often encountered in studies of amorphous and liquid materials is *g*(*r*). It is frequently called the pair distribution function by the liquids/amorphous community and the pair density function by the disordered crystalline materials community (Benmore, 2012 ▸). *g*(*r*) is functionally identical to the density function ρ(*r*); however, it has been scaled by the average number density, resulting in the relationship 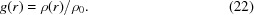
Note that the *g*(*r*) function is related to isothermal compressibility, defined in equation (9), via 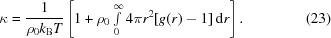
Up to this point, all described atomic PDFs have assumed a sum over all atom–atom pairs in a defined volume. However, one can define a ‘partial PDF’, *g*_μν_(*r*), which includes contributions from only those atoms in a given pair type. By definition, the sum of all possible partial PDFs will reconstruct the corresponding all-atom PDF. The most common convention requires that ‘Faber–Ziman partial structure factors’ be calculated for each atom pair. Yet other formalisms exist, each with their respective advantages. The Bhatia–Thornton formalism is an alternative representation of the system as the mean square fluctuations in the particle number, fluctuations in concentration and the correlation between these two correlations (Bhatia & Thornton, 1970 ▸). These can be, for a two-component system, directly mapped to Faber–Ziman using the equations in Bhatia and Thornton’s seminal 1970 work. The Ashcroft–Langreth formalism is another that is commonly used in theoretical and computational work owing to its connection to direct correlation functions (Ashcroft & Langreth, 1967 ▸). The mapping of the Ashcroft–Langreth to the Faber–Ziman equations is most readily accessible in equation 2.35 of the review paper by Fischer *et al.* (2005 ▸) The connection to the Faber–Ziman partial structure factors and both the total scattering structure factor and partial pair distribution functions is presented in Appendix *F* [App appf]. The weighted sum of the partial PDFs will result in a *g*(*r*) such that 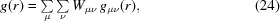
where *W*_μν_ is the associated weighting factor for the pair of atoms μ and ν. Note that *g*_μν_(*r*) is a ‘true’ distribution function as it does not include weighting by scattering lengths. However, this is not a distribution function in the statistical sense as the normalization is 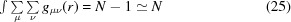
rather than one (McQuarrie, 2000 ▸). Different communities employ different normalization schemes for these weighting factors. Some communities will add an ‘*x*’ or ‘*n*’ superscript to *g*(*r*) to denote the weighting. Herein, we normalize them such that 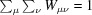 unless explicitly noted otherwise. For monatomic systems, the weighting factor is always one.

A form of confusion within the greater PDF community is the differences between the reduced pair distribution function, *G*(*r*), and the total radial distribution function, which is often also labeled *G*(*r*) (Keen, 2001 ▸). For clarity, we here refer to the total radial distribution function as *G*_K_(*r*). This form of the PDF is constructed from the sum of all partials, *g*_μν_(*r*), weighted according to concentration of atomic species, *c*, and associated coherent scattering power, *b*_coh_, such that 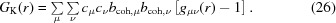
The relationship between *G*_K_(*r*) and *G*(*r*) is therefore 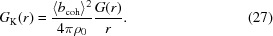



An important note, and an example of where confusion can occur for new practitioners, is that *G*_K_(*r*) in this work is equivalent to *G*(*R*) in the review paper of Fischer *et al.* (2005 ▸) This can be seen by comparing equation (27) in this work with equation 2.40 of Fisher *et al.*

A third variation commonly found in the crystalline PDF community, referred to as the differential correlation function, *D*(*r*) (Tucker *et al.*, 2007 ▸, 2017 ▸), is identical to *G*(*r*) apart from a constant scaling factor such that 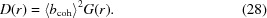
Related to the previous note about *G*_K_(*R*), *D*(*r*) here is different from the *D*(*r*) found in the review paper of Fischer *et al.* (2005 ▸) The *D*(*r*) in this work is equivalent to *G*(*R*) in the review paper of Fischer *et al.* This can be seen by comparing *G*(*r*) in equation (34) in this work with equation 2.26 of Fisher *et al.*

Another version of the PDF, primarily used in the liquids and glass community and referred to as the total correlation function, is *T*(*r*) (Soper, 1989 ▸; Hannon *et al.*, 1990 ▸). *T*(*r*) is related to *G*(*r*) as 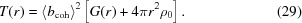



Additional minor variations of pair distribution function relationships can be found, but while some do surface occasionally in the modern literature, many are no longer actively utilized.

Figs. 3 ▸ and 4 ▸ graphically display examples of *R*(*r*), ρ(*r*), *g*(*r*), *G*_K_(*r*), *G*(*r*) and *T*(*r*) for the cases of crystalline MnO and liquid Ar, respectively. The inherent information content of all forms is the same. All PDFs show atomic pair–pair correlations as peaks centered at average pairwise distances in real space, with the height of these peaks informing on the frequency of these pairwise distances (often with the scattering power of atoms involved) and the widths related to the distribution of the pairwise distances. The functions feature different limiting behaviors at low and high *r*, summarized in Table 4 ▸. These limits, and the accompanying scaling of peak intensities as *r* increases, emphasize different features of interatomic order. Preferred usage has developed in various research communities according to some of these distinguishing behaviors.

The weighted radial distribution function, *R*(*r*), shown at the top of Figs. 3 ▸ and 4 ▸, and the radial distribution function, RDF(*r*), find limited use because they rapidly increase towards infinity with increasing *r*. Thus it is a challenge to visually inspect the local correlations on the same scale as the mid-to-long-range correlations.

In materials which lack long-range order, few important structural details exist at high *r*, and several functions are commonly used. We introduced definitions of pair distribution functions based on the density function, ρ(*r*), which is a straightforward quantity to calculate from atomistic simulations and models. It is shown in the panels second from the top in Figs. 3 ▸ and 4 ▸. *g*(*r*) is simply the number density divided by the average density, and it has found wide adoption in the amorphous and liquids community (Benmore, 2012 ▸). The limits of *g*(*r*) are absolutely defined to be zero prior to the first pair correlation and 1 at high *r*, which in practice can aid with data reduction and normalization procedures. An example of *g*(*r*) can be seen in the third panel from the top of Figs. 3 ▸ and 4 ▸. *G*_K_(*r*), shown fourth from the top in Figs. 3 ▸ and 4 ▸, shares many qualitative features with *g*(*r*), and while they appear nearly identical at first glance, they feature different units, limits and scaling behavior (refer to Appendix *A* [App appa], Figs. 3 ▸ and 4 ▸, and Table 4 ▸ for details).

To resolve certain details of local structure, researchers occasionally find it useful to preferentially weight a structural refinement towards features at low *r*, ignoring or downplaying longer-range features. While there is nothing implicitly wrong with this approach, this is a decision best applied at the time of modeling and stated clearly in analysis discussions. The issue with functions that inherently carry their own *r*-dependent weighting [such as *G*_K_(*r*) damping as 1/*r*] is that they require additional *r*-dependent normalization of residuals to uniformly treat misfit at different length scales. It can be argued that representations of the measured data should not themselves contain such an *r*-dependent feature bias.

The reduced pair distribution function, *G*(*r*) (second from the bottom in Figs. 3 ▸ and 4 ▸), is the most prevalent formalism used in the study of disordered crystalline materials and nanocrystalline materials and is the version of data compatible with the popular real-space PDF modeling program *PDFgui* (Farrow *et al.*, 2016 ▸). *G*(*r*) is also sometimes used in glass or molecular liquid studies, particularly when longer-range ordering is present. Arguably, the most advantageous feature of the *G*(*r*) formalism is that the amplitude of the oscillations is independent of *R* value. This means that the nature of a material’s structural coherence can be readily interpreted via visual inspection of *G*(*r*). It also means that residual differences between models and data are equally weighted at all *R* values.

It is sometimes asserted that *G*(*r*) is the most directly calculable function from experimental data (Egami & Billinge, 2012 ▸), as it is the direct Fourier transform of *S*(*Q*) and does not require any assumptions of number density or average scattering power. In practice, the data reduction procedures employed to generate *G*(*r*) typically involve a number of optimization steps, which effectively estimate various sample-dependent corrections either analytically (Peterson *et al.*, 2000 ▸; Jeong *et al.*, 2001 ▸; Qiu, Božin *et al.*, 2004 ▸) or via *ad* *hoc* methods (Neuefeind *et al.*, 2012 ▸; Juhás *et al.*, 2013 ▸).

*D*(*r*) shares many of the same features as *G*(*r*), as it is equivalent to 〈*b*_coh_〉^2^ *G*(*r*). Because of this subtle difference, there has been some confusion in the community about when to use *D*(*r*) compared with *G*(*r*) in different analysis methods. In practice, employing either *D*(*r*) or *G*(*r*) when using small-box modeling approaches (where a scale parameter for the data set can be freely refined) will produce identical model results. However, they cannot be used interchangeably in those methods that rely on absolutely normalized data, such as RMC-based modeling. This can be particularly tricky in those cases where 〈*b*_coh_〉^2^ is near to one, as the results of a large-box modeling approach may appear to be converging, but the results will be incorrect. Researchers are advised to carefully verify what form of the PDF they are employing, especially when using data from a new beamline (where data reduction protocols may differ) or employing a new form of analysis.

The neutron glass community tends to favor the *T*(*r*) formalism shown at the bottom of Figs. 3 ▸ and 4 ▸ (Hannon *et al.*, 1990 ▸; Ellison *et al.*, 1993 ▸). *T*(*r*) [and *G*(*r*)] scale relative to the number density as a function of *r*, as opposed to functions such as *g*(*r*) or *R*(*r*) (see Appendix *A*4). In the harmonic approximation of atomic motion, peaks are broadened symmetrically in *T*(*r*) [and *G*(*r*)] by thermal motions (Wright *et al.*, 1989 ▸), which is cited as a considerable advantage in differentiating between static and thermal disorder (Hannon *et al.*, 1990 ▸). This symmetry is noted by the glass community to lead to more accurate fitting for extracting coordination numbers (Benmore, 2012 ▸). However, *T*(*r*)’s overall *r*-dependent scaling means it is not practical for viewing wide ranges in real space, and thus it is not commonly used in studies of nanocrystalline or disordered crystalline materials.

Arguably, the density function is one of the most straightforward functions to calculate from an atomic model. We here present the conversions from this common quantity, ρ(*r*), and the other six common all-atom PDFs we have discussed herein. Table 5 ▸ provides conversions between some of the real space functions. The full list of conversions originating from each function in turn can be found in Appendix *A* [App appa].

**Figure 5:**
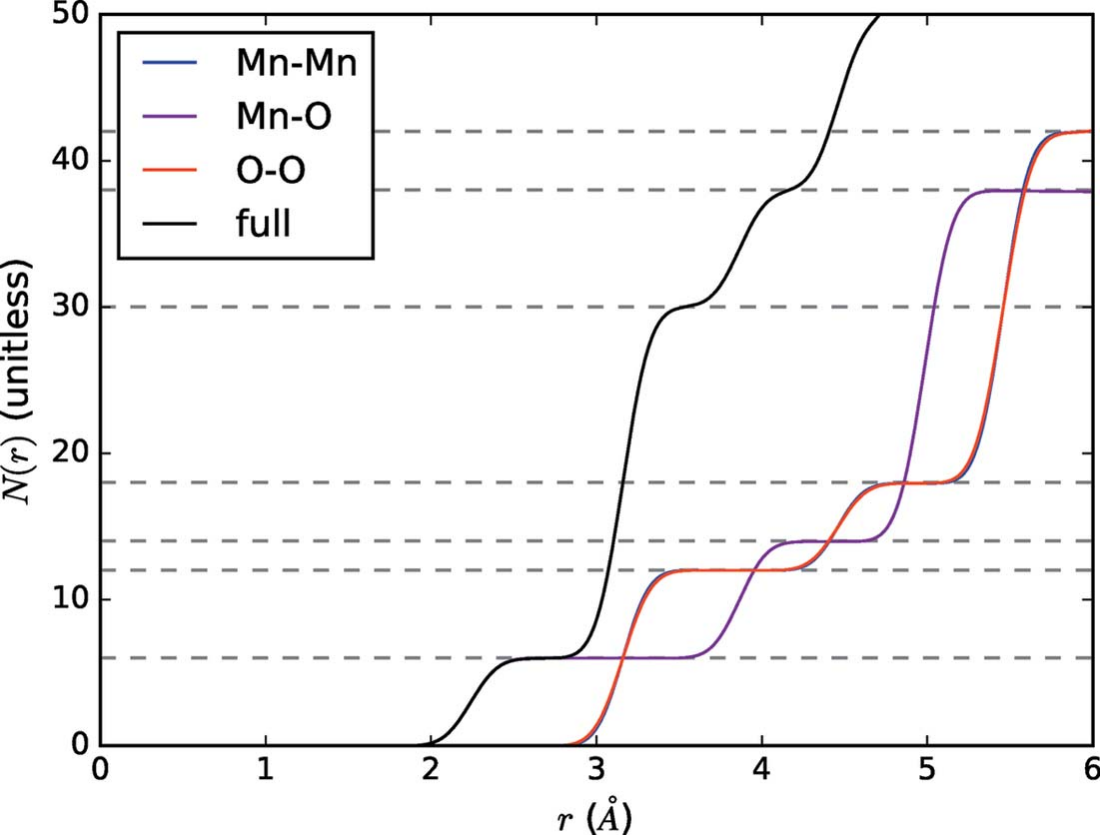
Accumulated correlation number for MnO plotted with its partials. The partial and full coordination numbers are marked with dashed lines. Note that the partial coordination numbers for Mn–Mn and O–O are identical owing to symmetry, but the isotropic displacement parameters, *U*_iso_, are different, which is visible near the plateaus in the Mn–Mn and O–O partials.

Finally, PDF data are often interpreted using the coordination number, *N*(*r*). This is the number of atoms between *r*_min_ and *r*_max_. The coordination number is described mathematically in terms of the partial *g*_μν_(*r*) functions (Soper, 2010 ▸):
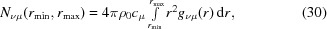
which explicitly does not include the scattering lengths. A closely related function is the accumulation of this summed over all atoms: 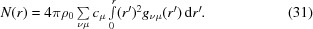
This quantity is shown in Fig. 5 ▸. Note that coordination number is not weighted by scattering length, such that it cannot be easily transformed to other real-space functions without employing partial PDF functions.

## 5. Summary   

This paper has provided a resource to understand the relationships of and convert between eight real-space pair distribution functions commonly found in the scientific literature. This purely instructional work offers direct mathematical conversions, graphical representations and a practical discussion of function characteristics, meant as an updated step-by-step reference for new practitioners and those seeking to compare their results with those of other researchers. While the appearance and weighting of these representations can vary, often to emphasize certain features of interest, the inherent structural information must be the same among these different forms. Although we respect the decisions of individual researchers to use whichever PDF formalism they find most appropriate, we encourage convergence and standardization where possible. On the basis of the comparison and discussion presented herein, we endorse the use of two primary formalisms: *G*(*r*) and *g*(*r*). The reduced pair distribution function, *G*(*r*), is recommended because of its uniform weighting at all *R* values. It has been broadly employed by the disordered crystalline and nanocrystalline communities. In contrast, the pair distribution function, *g*(*r*), is recommended as it is conveniently bounded at 0 and 1 (simplifying normalization procedures), features symmetric peaks and emphasizes low-*r* features. Thus it has been widely adopted by the liquids, amorphous and glass communities.

We also encourage that, when reporting a PDF, authors overtly define which real-space distribution functions they present. Reciprocal-space functions should be described in terms of *S*(*Q*) (which has an agreed upon and consistent definition across fields). For example, ‘we show the structure function, *F*(*Q*) = *Q*[*S*(*Q*) − 1]’. Similarly, real-space functions should be described in terms of ρ(*r*). For example, ‘we fit the pair distribution function, *G*(*r*) = 4π[ρ(*r*) − ρ_0_]’. It is our hope that the derivations, tables and figures presented in this work help serve as a reference tool for researchers to easily navigate the PDF landscape and guide towards a convergence of total scattering data formalisms.

## 

## Figures and Tables

**Figure 6:**
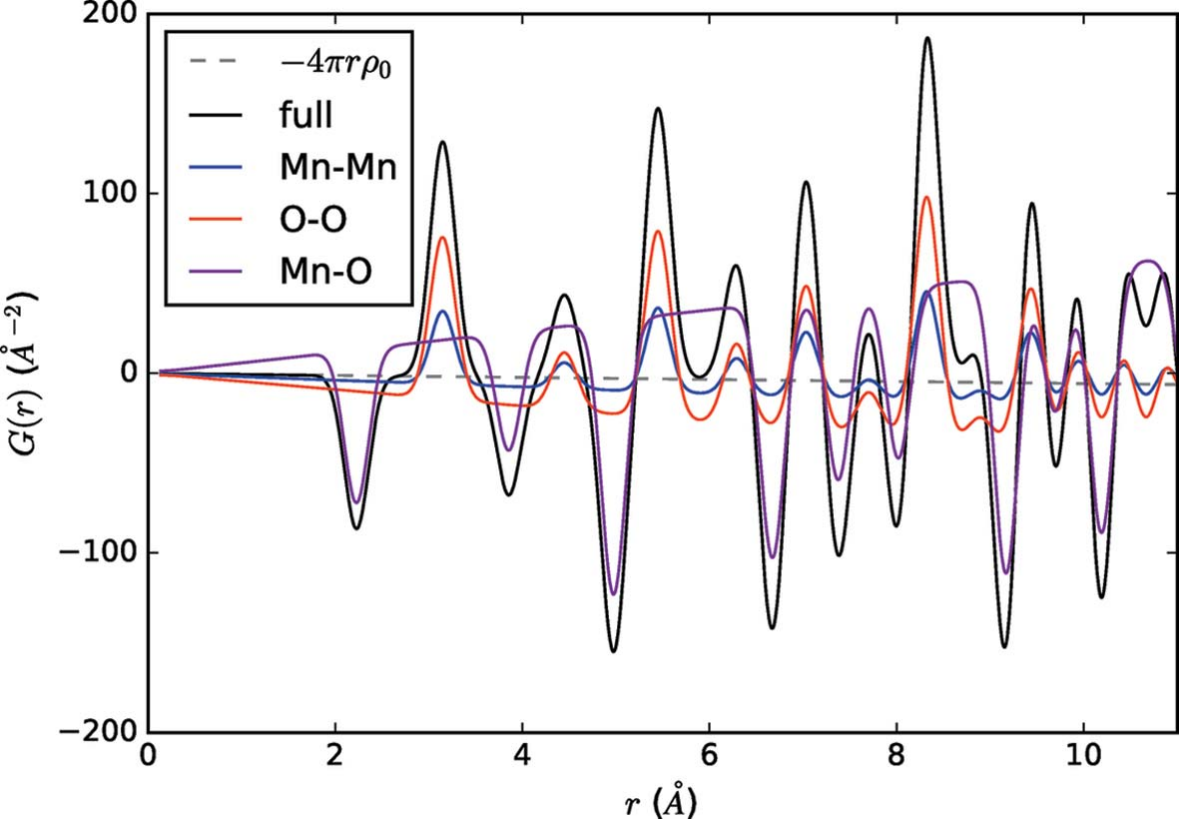
Partial reduced pair distribution functions for MnO.

**Table 1:** Summary of structure of MnO used for examples (Sasaki *et al.*, 1979 ▸) The structure is 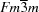 with a lattice constant of 4.446 (1) Å.

Atom	(*x*, *y*, *z*) (fractional units)	*B*_eq_ (Å^2^)
Mn^2+^	(0.0, 0.0, 0.0)	0.617 (5)
O^2−^	(0.5, 0.5, 0.5)	0.72 (1)

**Table 2:** η for the materials chosen at room temperature (300 K) The bulk modulus for MnO is taken from the article by Zhang (1999 ▸).

Material	*K*_0_ = 1/κ (GPa)	ρ_0_ (atoms Å^−3^)	η
Ar	0.552	0.02138	0.046
MnO	148	0.0455	0.0013

**Table 3:** Limits of reciprocal-space functions Although atoms are normally not included in the unit definition, they are included here for clarity.

Function	Low-*Q* behavior	High-*Q* behavior	Units
*I*(*Q*)	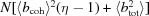	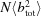	Barn atom
*S*(*Q*)	η	1	Unitless
*F*(*Q*)	(η − 1)*Q*	0	Å^−1^
*F*_K_(*Q*)	〈*b*_coh_〉^2^(η − 1)	0	Barn

**Table 4:** Limits of real-space functions For materials with long-range order (*i.e.* crystalline), the high-*r* behavior is often obscured by the peaks in the distribution function. Like radians, atoms is normally not listed as a unit, but this table explicitly mentions it for added clarity.

Function	Low-*r* behavior	High-*r* behavior	Units
*R*(*r*)	0	4πρ_0_ *r* ^2^	Atom Å^−1^
ρ(*r*)	0	ρ_0_	Atom Å^−3^
*g*(*r*)	0	1	Unitless
*G*_K_(*r*)	−〈*b*_coh_〉^2^	0	Barn
*G*(*r*)	−4πρ_0_ *r*	0	Atom Å^−2^
*D*(*r*)	−4πρ_0_〈*b*_coh_〉^2^ *r*	0	Barn atom Å^−2^
*T*(*r*)	0	4πρ_0_〈*b*_coh_〉^2^ *r*	Barn atom Å^−2^
*N*(*r*)	0	∝ *r* ^3^	Atom

**Table 5:** Conversions to and from ρ(*r*) A complete list of conversion factors is given in Appendix *A* [App appa].

Formalism in terms of ρ(*r*)	ρ(*r*) in terms of formalism
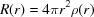	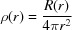
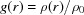	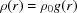
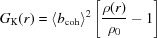	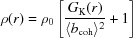
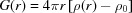	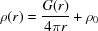
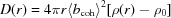	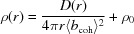
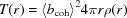	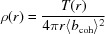

**Table 6:** Description of subscripts, *i*, for measured intensities, *I*_*i*_(*Q*), and differential cross sections, 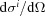

Subscript	Term
s	Sample
c	Container
a	Apparatus
sc	Sample + container
sca	Sample + container + apparatus
e	Background / empty diffractometer
n	Normalization

**Table 7:** Correction terms for the different types of experimental measurement setups, including absorption, multiple scattering and inelastic recoil corrections The μ and ν terms are defined in Table 6 ▸.

Correction term	Definition
*A*_μ,ν_	Absorption correction factor for scattering in μ absorbed by ν
*M*_ν_	Multiple scattering correction factor for ν
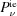	Inelastic recoil correction factor for ν

**Table 8:** The different experimentally measured intensities required for a total scattering experiment

Experimental term	Definition
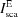	Experimentally measured intensity of sca
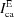	Experimentally measured intensity of ca
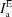	Experimentally measured intensity of a
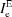	Experimentally measured intensity of e
Φ	Normalization term to account for detector efficiency, solid angle coverage *etc*.

**Table 9:** Table of scattering lengths for the example materials taken from Sears (1992 ▸)

		*b*_coh_ (fm)		
Atom	*b*_tot_ (fm)	Real	Imaginary	Length
Ar	2.3313	1.909	0.0	1.909
Mn	4.1363	−3.73	0.0	3.73
O	5.8032	5.803	0.0	5.803

**Table 10:** Calculated values for the normalized Laue term shown with fixed precision Note that the normalized Laue term is not necessarily zero for monatomic materials.

Material	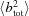 (fm^2^)	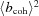 (fm^2^)	*L*
Ar	5.435	3.644	0.491
MnO	25.393	1.074	22.636

**Table 11:** Weights, *W*_νμ_, for partials for the materials chosen The atom listed first in the chemical formula is α and the second is β.

Material	*W*_αα_	*W*_αβ_	*W*_ββ_
Ar	1.000	N/A	N/A
MnO	3.238	5.037	7.836

## Appendix A Conversions between real-space functions

This appendix will provide transformations between the various real-space functions. Several notes are needed before the listing of equations. First, we will assume a shape function of γ_0_(*r*) = 1. This is true for sufficiently bulk material measurements (*i.e.* material structures exhibit translational periodicity and thus do not have finite size, shape or morphology effects contributing to the experimental PDF). Second, the correlation number, *N*(*r*), will not be mentioned since it can only be correctly calculated from the partial distributions. Note that the rise of *N*(*r*) at the location of an isolated peak corresponds to the coordinate number of that pairwise peak. Third, the normalization of *g*(*r*) provided in the main text is applicable here.

The original purpose of this appendix was to provide simple recipes for fellow researchers to transform their data or model calculations between the real-space forms. An additional benefit of generating this appendix, as well as the limits in Tables 3 ▸ and 4 ▸, was further validation that the conversions between conventions and the presentation of the conventions themselves are correct.

## Appendix B Details of liquid argon molecular dynamics simulations

For all Ar simulations, the system consisted of a cubic simulation cell with 50 000 atoms and a velocity-Verlet-like (Verlet, 1967 ▸) time integrator was used with a 1 fs timestep. A Lennard–Jones pair potential with a 15 Å cutoff was used with a tail correction applied, with ε = 0.238067 kcal mol^−1^ and σ = 3.405 Å (Yarnell *et al.*, 1973 ▸). Initially, the atoms were randomly placed in the box and then force minimized with a force tolerance of 10^−8^ kcal (mol Å)^−1^. To produce the real- and reciprocal-space patterns, canonical (NVT) ensemble simulations were carried out with *T* = 86.56 K and ρ_0_ = 0.02138 atoms Å^−3^ to reproduce previous results (Yarnell *et al.*, 1973 ▸). A Nosé–Hoover-style thermostat (Nosé, 1984 ▸; Hoover, 1985 ▸) was used to keep constant temperature with a relaxation time of 0.1 ps. For the isothermal compressibility calculations, isobaric isothermal (NPT) simulations were carried out at the same temperature as the NVT simulation but with different pressures. A Nosé–Hoover-style barostat (Nosé, 1984 ▸; Hoover, 1985 ▸) was used to keep constant pressure with a relaxation time of 1 ps. The PDF was then calculated over a real-space range 0.075 ≤ *r* ≤ 100 Å with a bin width of δ*r* = 0.05 Å. The reciprocal-space data were generated through inverse Fourier transform. The reciprocal-space data are scaled such that forward Fourier transforms recreate the original PDFs precisely.

## Appendix C From experimentally observed scattering intensities to differential cross sections

The authors mostly ignored the steps involved in reducing data from measured scattering intensities (Section 3) to reciprocal-space functions in the main text. This appendix will shed light on some of the corrections necessary to transform measured intensities to a single scattering differential cross section (DCS), , which can then be used in equation (4). For Rietveld analysis, this level of rigor for corrections is not usually needed for most samples as these effects are frequently addressed as part of the measurement’s ‘background intensity’, which is normally fitted with a polynomial to reduce its influence on the profile refinement. However, for real-space total scattering measurements, these ‘minor’ effects can produce a signal comparable to the reciprocal-space features of local disorder and therefore must be accounted for.

Repeating equation (4),

where *I*(*Q*) is the scattering from the sample (using the same convention as Rietveld). Its relation to the differential cross section (DCS) is simply (Egami & Billinge, 2012 ▸)

where *N* = ρ_0,eff_ *V* is the number of atoms illuminated, *V* is the illuminated volume and ρ_0,eff_ is the effective number density as described in Appendix *E* [App appe]. Because corrections are easier to explain in terms of the DCS, we rewrite equation (4) as

The DCS can only be found by fully correcting the measured intensity. Proper treatment of total scattering data requires correcting for effects such as attenuation through the sample material and its environment, subtraction of multiple scattering events, and recoil or inelastic effects. That being said, the notations for the various corrections do not have a standard set of definitions. The associated measurements for these corrections (*e.g.* empty container, empty sample environment, normalization) are no longer as unusual to perform as they used to be. Table 6 ▸ describes the standard set of measurements required and associates subscripts to refer to them. Additionally, Paalman & Pings (1962 ▸) developed a detailed notation for the attenuation correction terms which will be used here. Table 7 ▸ describes the notation for the correction terms applied to the experimental measurements.

For the measurement of the sample and container combined there are four terms of scattering and attenuation (also called absorption) in the experiment. For example, the scattering that occurs in only the sample but is attenuated by both the sample and container component is denoted as *A*_s,sc_. Similarly, the multiple scattering is denoted as *M*_μ_ for the μth measurement, and thus the sample and container are denoted *M*_sc_. Finally, the inelastic recoil correction is denoted as  for the νth species; thus the container inelastic correction would be .

Having the various functions and subscript definitions in hand, the description of what is actually experimentally measured, , can be introduced. The experimentally measured intensities are summarized in Table 8 ▸.

These experimentally measured intensities are represented by the functional forms below with the necessary correction terms included from Table 7 ▸:

Note that the double-differential cross-section term appears in these equations, with ℏω being the energy loss. For the ultimate goal of producing a suitable reciprocal-space function, one must integrate over ω to obtain the energy-integrated DCS. If all scattering were purely elastic from the sample, we would simply have

Yet, there are undoubtedly inelastic scattering events that occur and necessary terms must be included in the above equation for the specific experimental technique. The equation for an experimental integration over ω is given as

where β(*k*_i_, *k*_f_) accounts generally for terms such as detector efficiency, incident spectrum and other terms needed based on the nature of the experimental method (*i.e.* constant wavelength or time of flight).  is the inelastic correction term that occurs due to both the recoil of the atomic nuclei from the neutron collision and, for time of flight, the fact that the measurement is carried out at a fixed angle and not fixed momentum transfer, *Q* (Soper, 2009 ▸). For the functional forms of β(*k*_i_, *k*_f_) for constant wavelength or time of flight, we refer the reader to reports in the series by Powles (1973 ▸, 1978*a* ▸,*b* ▸) or the *GUDRUN* manual (Soper, 2010 ▸).

Real-space techniques are often colloquially referred to as ‘total scattering’ because they use all of the reciprocal-space information rather than just the Bragg peaks. As mentioned in the main text, this term has a second meaning when it is applied to describe integration over energy transfer. This is not the same as the elastic scattering, which can only be properly measured when the incident and final energy of the probe are known. Integrating the energy yields the following set of equations:

Solving each of the equations above for the respective DCS [and simplifying  to just ] provides

Reducing the equations into terms that only involve the corrections and the measured intensities, the sample DCS is

the container DCS is

and the sample environment apparatus DCS is

Note that here the normalization term, Φ, has not been made explicit. Thus, an additional experimental measurement is needed to characterize the normalization term. Using our definitions in equations (45), we can define this normalization measurement as

The normalization measurement performed will differ according to the probe used, X-rays or neutrons. Yet, both serve a similar purpose in that they factor out the dependence of detector efficiencies, solid angle coverage *etc*. As a specific example, vanadium is often utilized for neutron scattering, mainly because it has a small coherent scattering length, implying that the distinct scattering signal is small compared with the self-scattering and thus it exhibits a relatively smooth diffraction pattern. Since self-scattering does not vary much with *Q* or 2θ, the differential cross section is essentially , where *b*_tot,v_ is the total scattering length of vanadium from equation (53). For more detail on reasons to use vanadium, we refer the reader to Section 3.8.1 of the *GUDRUN* manual (Soper, 2010 ▸) and for more information regarding the details of the normalization, we refer the reader to both Section A5 of the text by Egami & Billinge (2012 ▸) and Section 3.8 of the *GUDRUN* manual (Soper, 2010 ▸). By substituting 〈〉 into equation (50) for dσ^n^/dΩ and the  term from equation (45), we can derive the following:

Solving for Φ gives

which defines the final Φ term needed in equations (47), (48) and (49).

Solving equation (47) from the experimentally measured intensities then allows one to arrive at the differential cross section, also commonly defined as *I*(*Q*)/*N* in the total scattering community. This is the same as that defined in equation (1) and throughout the rest of this work. For further details on the data reduction described briefly herein and all necessary corrections, the reader is referred to more complete discussions in the literature (Howe *et al.*, 1989 ▸; Hannon *et al.*, 1990 ▸; Soper, 2010 ▸; Egami & Billinge, 2012 ▸). There are also other excellent references that deviate in detail from the reduction described herein, specifically in the steps where corrections are applied. (Windsor, 1981 ▸) Naturally, refinement of the methodology has occurred with time.

The derivations herein integrated the scattering intensities over all measurement energies, where inelastic and multiple scattering effects are handled through data corrections. Several authors have noted the implications of this simplification (Page *et al.*, 2011 ▸). Dynamic PDF has emerged as an extension of total scattering, making explicit use of the energy dependence (Egami & Billinge, 2012 ▸). This falls outside the scope of the present work.

## Appendix D Calculating the normalized Laue term

There is often confusion when determining the normalized Laue term of equation (5). Lovesey (1986 ▸) introduced quantities related to the total cross section of the material,

and the coherent cross section,

Here, the subscript coh stands for coherent and tot stands for total, where  must be inferred from the total cross section (Sears, 1992 ▸). In the Faber–Ziman scheme, 〈*b*_coh_〉^2^ comes from the sum of the partials and the pupose of the  term is to remove the self-scattering from the differential cross section (Faber & Ziman, 1965 ▸). These are the terms that appear in the normalized Laue term with the factor of 4π canceled out. Note that 〈σ_coh_〉 ≠ 4π〈*b*_coh_〉^2^, but Lovesey’s notation obscures this fact. Being more explicit, it is straightforward to calculate the terms as originally intended. First we introduce a normalized concentration

where *N*_α_ is the number of atoms of type α and . This provides for the normalization that . The two quantities needed in the normalized Laue term are simply

and

This formulation of 〈*b*_coh_〉^2^ makes more explicit that the complex scattering lengths, with sign, are averaged. Sears (1992 ▸) offers a useful discussion of calculating average cross sections. Unique to neutron measurements, this can lead to materials where the normalized Laue term becomes infinite because of atoms with a negative scattering length. Ti_2.08_Zr is a classic example of 〈*b*_coh_〉^2^ = 0 and .

An additional complication is found when  is calculated. Since 1 barn = 100 fm^2^, people often calculate  by simply dividing by 4π and ignoring the units. While the factor of 100 will cancel out in the normalized Laue term, the values of the individual terms will be listed in units of 10 fm (or dekafemtometres) rather than fm. Tables 9 ▸ and 10 ▸ show calculations for the example materials.

## Appendix E Determining number density

Often when processing or analyzing data, the effective number density, or packing fraction, is treated as an adjustable parameter. Here we demonstrate how to calculate it and give guidance on reasonable limits. The effective number density affects absorption and multiple scattering corrections as well as the total number of illuminated atoms as described in Appendix *C* [App appc]. Since it is difficult to directly measure the packing fraction precisely, one can see why it is often tuned during data reduction.

First we introduce the concept of effective number density,

which is based on the crystallographic number density, ρ_0_, using a packing fraction *f* ∈ (0, 1]. For measurements of crystalline powders, the packing fraction rarely exceeds *f* = 0.5. The number density for a crystalline sample is often phrased in terms of the number of atoms per unit cell, called the *Z* parameter, and the unit-cell volume. Using standard crystallographic conventions (Giacovazzo, 1992 ▸)

For liquids, ρ_0_ is not normally known. The technique for establishing it is to calculate ρ_0,eff_ from the mass density, ρ_m_. This is done by weighing the empty sample container, adding the sample, and then weighing the filled container and measuring how full the container is. This measurement will give a mass density with the packing fraction correctly accounted for. Then the effective number density is

where *N*_A_ is Avogadro’s number, *m*_α_ is the atomic mass, and *c*_α_ is the normalized concentration of element α or the atomic fraction as defined in Appendix *D* [App appd]. This procedure is often used for crystalline samples as well. An alternative to this approximation is to measure the volume by displacement in a fluid that the material does not react with and is insoluble within. However, it is likely to be difficult to recover a sample from this type of measurement so it is recommended that it is done after the scattering measurement is completed.

## Appendix F Partial structure functions

To use the prevalent Faber–Ziman partial structure functions, one must first define weights (Faber & Ziman, 1965 ▸; Suck *et al.*, 1993 ▸; Egami & Billinge, 2012 ▸)

which are normalized such that . Note that the same symbol is used with an alternative normalization in some communities with . The Faber–Ziman partial structure functions are related to the total scattering structure factor

with *A*_νμ_(*Q*) = *S*_νμ_(*Q*) in the nomenclature that Egami uses. The benefit of this normalization of *S*_νμ_(*Q*) is that it has the same asymptotes as *S*(*Q*) with

and

In equation (61), 〈*b*_coh_〉^2^ is the average scattering length for the material and the numerator is the average scattering length for the atoms contributing to the partial.

Then it is straightforward to define a partial reduced pair distribution function as

which observes a summation rule of

The other real-space correlation functions follow analogous forms.

To clarify a related point, difference correlation functions are a distinct concept. Instead of being the correlation of two atomic species, it is all correlations with an atom of type ν at the origin. In other words

which follows the summation rule

A more detailed description is given by Egami & Billinge (2012 ▸, Sections 3.1 and 3.2).

For a two-atom system with atoms labeled α and β, the summation rule for the weights in equation (61) is

For a monatomic system, there are no partial functions. See Table 11 ▸ for explicit calculation of the values. Fig. 6 ▸ shows the partial *G*(*r*).
