# Illustrated formalisms for total scattering data: a guide for new practitioners. Corrigendum and addendum

**DOI:** 10.1107/S1600576721007664

**Published:** 2021-09-13

**Authors:** Peter F. Peterson, David A. Keen

**Affiliations:** a Computer Science and Mathematics Division, Oak Ridge National Laboratory, Oak Ridge, TN, USA; b Neutron Scattering Division, Oak Ridge National Laboratory, Oak Ridge, TN, USA; c ISIS Facility, Rutherford Appleton Laboratory, Harwell Campus, Didcot, Oxfordshire, United Kingdom

**Keywords:** total scattering, pair distribution function

## Abstract

Errors and ambiguities in the article by Peterson, Olds, McDonnell & Page [*J. Appl. Cryst.* (2021), **54**, 317–332] are corrected and clarified, respectively.

In the article *Illustrated formalisms for total scattering data: a guide for new practitioners* (Peterson *et al.*, 2021 ▸) the authors provide a detailed comparison between the various notations and formalisms used by the total scattering/pair distribution function (PDF) community. The paper repeats the relationships already established by Keen (2001 ▸), presents look-up tables for easy conversion between functions, and provides graphical examples based on calculated neutron scattering functions of liquid argon and powdered MnO from molecular dynamics simulations and the cubic crystal structure, respectively. However, there is a confusing choice of units when some of the functions are presented graphically, leading to a mis-labelling the *y* axes of several of the figures. Furthermore, the low-*Q* limits have been defined incorrectly, such that they are only applicable for monoatomic materials. Given the pedagogical nature of the article (Peterson *et al.*, 2021 ▸), we felt it necessary to provide this corrigendum to clarify any unintended confusion. For greater clarity, we provide a worked example based on experimental GEM data from BaTiO_3_ (Senn *et al.*, 2016 ▸) in support of these revisions.

As already pointed out by Keen (2001 ▸) and Peterson *et al.* (2021 ▸), in this field different communities lay claim to the same function names for subtly different definitions. In order to be completely explicit in this corrigendum we will use the subscript ‘K’ and superscript ‘PDF’ to clarify multiply defined functions consistent with the sub/superscripting used by Peterson *et al.* (2021 ▸) and Keen (2001 ▸), respectively. We have not assigned these additional labels to functions [such as *S*(*Q*) and ρ(*r*)] that are defined identically by Keen (2001 ▸) and Peterson *et al.* (2021 ▸). This somewhat tedious notation will be helpful when presenting clarifying points and is detailed in Table 1 ▸.

Neutron scattering lengths were typically given in units of 10^−12^ cm, *i.e.* 10 fm units, a natural working unit since neutron cross sections are expressed in barns (10^−24^ cm^2^) [for example *International Tables for X-ray Crystallography*, Vol. III (1962)]; the powers of ten, although implicitly present, could be ignored in practice. They are now more commonly tabulated in fm (Sears, 1992 ▸) and these are the values used by Peterson *et al.* (2021 ▸). This has introduced an inadvertent scaling error in Figs. 1(*e*), 1(*f*), 2(*e*), 2(*f*), 3 [*G*_K_(*r*) and *T*(*r*)] and 4 [*G*_K_(*r*) and *T*(*r*)] of Peterson *et al.* (2021 ▸); the functions in the figures with *y* axes labelled ‘barn’ or ‘barn/Å^2^’ should be 100× smaller than presented therein. Those functions that are further scaled by 〈*b*_coh_〉^−2^ [*e.g.* *F* ^PDF^(*Q*), *G* ^PDF^(*r*) or ρ(*r*)] are unaffected as the scattering length units cancel out.

The low-*Q* limit of total scattering structure factors is related in a complex way to thermodynamic functions and fluctuations [see for example Table 30.2 of Cusack (1987 ▸)] most of which, for ‘well behaved’ non-magnetic systems, are assumed to give a small correction to the expected level. It can be further complicated by other sources of low-*Q* ‘small-angle’ scattering, such as from longer-ranged scattering density variations (*e.g.* from nanoparticles) and magnetism, all of which are ignored in the following derivations. The low-*Q* limit of the partial total scattering structure factors, *A_ij_* (*Q*), is given in equation (13) of Keen (2001 ▸) and by McGreevy & Mitchell (1982 ▸) and is frequently shown in experimental data (Fischer *et al.*, 2006 ▸; Bowron *et al.*, 2006 ▸):
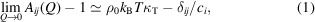
where the symbols have their usual meanings [*e.g.* in the text following equation (8) of Peterson *et al.* (2021 ▸)]. The first term on the right-hand side of equation (1) is the isothermal compressibility term, η, of Bhatia & Thornton (1970 ▸). It is usually small relative to the other terms in the low-*Q* limit of the total scattering structure factors (given below) and frequently treated as zero for data normalization purposes (although note that this may not always be the case and it may provide useful physical insight; Cusack, 1987 ▸). *A*_*ij*_(*Q*) is defined identically by Keen (2001 ▸) and Peterson *et al.* (2021 ▸). Since 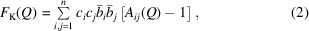
this gives rise to the following low-*Q* limits for the total structure factors, 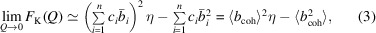
and with *S*(*Q*) = *F*_K_(*Q*)/〈*b*_coh_〉^2^ + 1 yields 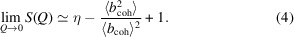
Here we have explicitly propagated η through the equations above, rather than using it more flexibly [as was done by Keen (2001 ▸)], whilst still bearing in mind that the definition in equation (1) is not valid in all circumstances (Cusack, 1987 ▸). These limits are different from those given in equations (7) and (64) and Table 3 of Peterson *et al.* (2021 ▸), which are only valid for monatomic systems. The corrected behaviour for various total scattering structure factors is summarized in Table 2 ▸, and the limits are recalculated using the average neutron scattering length constants in Table 3 ▸ to give the results in Table 4 ▸. Taking these points together, and as an example, we show in Fig. 1 ▸ a corrected version of Fig. 1 ▸ of Peterson *et al.* (2021 ▸) with very different low-*Q* limiting values and a much-reduced *y*-axis scale for *F*_K_(*Q*). Fig. 2 and the plots of *G*_K_(*r*) and *T*(*r*) in Figs. 3 and 4 in the original article need to be similarly modified but are not included here.

The above discussion also highlights another important point. Although the scaled functions [*S*(*Q*), *F* ^PDF^(*Q*) and *G* ^PDF^(*r*) *etc*., which are divided through by 〈*b*_coh_〉^2^], are useful when comparing with models and calculations, the functions *F*_K_(*Q*), *G*_K_(*r*) *etc*., which are not scaled by 〈*b*_coh_〉^−2^, permit a much more direct and unambiguous assessment of absolute data normalization when correcting experimental data. *F* ^PDF^(*Q*) and *G* ^PDF^(*r*) are more challenging in this regard as their respective *Q*- and *r*-dependent asymptotes to the origin make determination of the low-*Q* and low-*r* trends less obvious ‘by eye’. *S*(*Q*) should also be used cautiously; although it is unitless this hides the fact that scattering factors are incorporated within the function, and even though many *S*(*Q*) have a low-*Q* limit that is close to zero this does not mean that zero is the limiting value by definition.

As a worked example of this, we show in Fig. 2 ▸ data from BaTiO_3_ measured on GEM (Hannon, 2005 ▸) at 15 K (Senn *et al.*, 2016 ▸), which have been corrected using the *Gudrun* program (Hannon *et al.*, 1990 ▸; Soper, 2017 ▸). The Ti atoms in BaTiO_3_ have a negative neutron scattering length and the relevant average scattering constants are listed in Table 3 ▸. The high- and low-*Q* levels of the corrected differential scattering cross section (they should equal 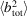 and tend to ∼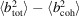 = 0.048, respectively) are immediate indicators of the quality of the data correction [as are the limits of *F*_K_(*Q*), *i.e.* after subtraction of 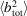; see Fig. 2 ▸(*a*)]. Typically when correcting data, a ‘good’ low-*Q* limit is often much harder to achieve than a ‘good’ high-*Q* limit. This is especially the case for time-of-flight neutron diffractometers where data corrections are usually more challenging in the lower-*Q* regime. The low-*Q* limit of *S*(*Q*) should approximately equal 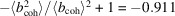 [Fig. 2 ▸(*c*)]. The low-*r* levels of *G*_K_(*r*) = −〈*b*_coh_〉^2^ and *g* ^PDF^(*r*) = 0 [Figs. 2 ▸(*b*) and 2 ▸(*d*), respectively]. Here a back-transform correction has been applied to direct these PDFs to their theoretical values for *r* < 1 Å; encouragingly these values are maintained to much higher *r*, including in the gaps between the first few low-*r* peaks. For completeness, plots of *F* ^PDF^(*Q*) and *G* ^PDF^(*r*) are shown in Figs. 2 ▸(*e*) and 2 ▸(*f*), respectively.

Finally, we note an inconsistency in the discussion of symmetric PDF peaks by Peterson *et al.* (2021 ▸). The different *r* dependencies mean that it is not possible for all definitions of the PDF function to show symmetric peaks centred at the average pairwise distances. A symmetric and centred peak in *G* ^PDF^(*r*) will not be symmetric and centred in *g* ^PDF^(*r*) [equivalent to 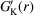 of Keen (2001 ▸)]. Symmetric peak fitting should only be carried out using PDF functions such as *G* ^PDF^(*r*) or *D*(*r*) (Olds *et al.*, 2018 ▸).

We have worked together on this corrigendum to try to ensure that these corrections to the Peterson *et al.* (2021 ▸) paper are clear and that the explicitly labelled functions herein mean that the relational expressions first established by Keen (2001 ▸) are not confused by the subtly redefined functions presented by Peterson *et al.* (2021 ▸), thus undermining the purpose of both papers. Total scattering notation has evolved over time since the equations were first conceived of by Zernike and Prins in 1927 (see a recent review and references therein; Keen, 2020 ▸), but it has stabilized over the past 20 years within the now mature total scattering community, in part through the cross-referencing of equations following Keen (2001 ▸). We as a community have a responsibility to ensure that we do not further compound any perceived notational confusion we might be trying to mitigate. Hopefully Keen (2001 ▸) and Peterson *et al.* (2021 ▸) with this corrigendum article will continue to provide the necessary clarity in total scattering function definitions.

## Figures and Tables

**Figure 1:**
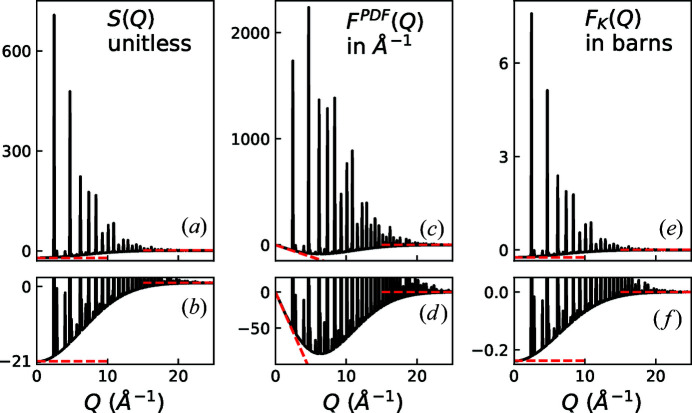
Redrawn version of Fig. 1 ▸ of Peterson *et al.* (2021 ▸) with corrected limiting values and scaling. The figure shows the comparison between various reciprocal-space neutron total scattering data from MnO: (*a*), (*b*) *S*(*Q*); (*c*), (*d*) *F* ^PDF^(*Q*); and (*e*), (*f*) *F*_K_(*Q*). The upper plots show an overview of the various functions, although the height of the Bragg peaks is mostly dictated by the width of the resolution function used (the same width was used in all plots). The asymptotes at high and low *Q* are highlighted with red dashed lines and pertinent values are given in Table 4. The low-*Q* limit of *F* ^PDF^(*Q*) is a sloped line which only remains linear for an extended range of *Q* because these are synthetic data with infinite sample size (and no magnetic contribution). However, the same behaviour holds true for highly ordered BaTiO_3_ in Fig. 2. Note that contributions from the terms involving η for MnO are effectively zero on the scale of these figures.

**Figure 2:**
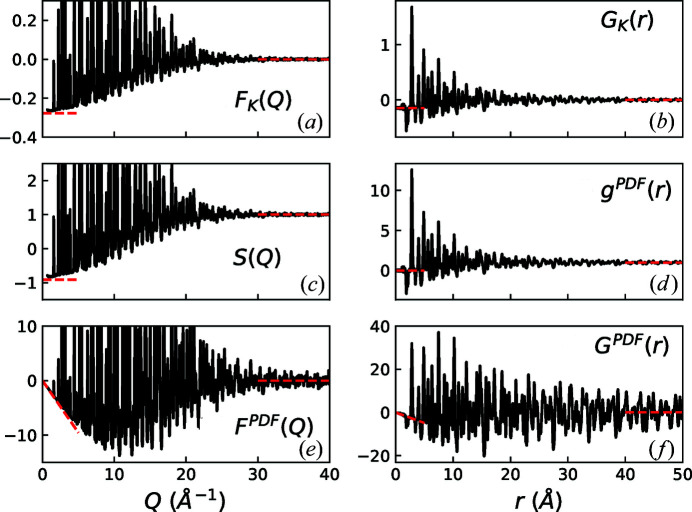
Experimental neutron total scattering functions from BaTiO_3_ at 15 K (Senn *et al.*, 2016 ▸), using ρ_0_ = 0.0779 atoms Å^−3^, determined from Rietveld refinement of the data. The various functions are defined using the equations given in Table 1 ▸. The asymptotes at high and low *Q* (or *r*, as appropriate) are highlighted using red dashed lines with values for reciprocal-space functions from Table 4 ▸; all real-space function limits are clearly seen to be 0 or 1 except for the low-*r* limit of *G*_K_(*r*), which is −〈*b*_coh_〉^2^ = −0.145 barn.

**Table 1:** Connection of formalisms to those found in previous work It is fully expected that future publications will not employ the subscript/superscript used here, but might nonetheless refer to these specific equations when establishing notation or define equations in terms of *S*(*Q*) or ρ(*r*) which have common meaning.

Function	Peterson *et al.* (2021) equation	Keen (2001 ▸) equation
*F*^PDF^(*Q*)	10	45 (implicit)
*S*(*Q*)	4	19/20
*F*_K_(*Q*)	11/13	9/19

*G*^PDF^(*r*)	19	43
*g*^PDF^(*r*)	22	41
ρ(*r*)	16	46
*G*_K_(*Q*)	26/27	10/44

**Table 2:** Limits of reciprocal-space functions This is a corrected version of Table 3 of Peterson *et al.* (2021 ▸) with all low-*Q* limits changed and monatomic low-*Q* limits added. The ‘low-*Q* limit’ column is correct for all materials (noting that these expressions are only approximately followed for measurements of real materials and assume there is no additional ‘small-angle’ scattering) and the ‘monatomic low-*Q* limit’ largely replicates the values in the original article. For solids and many liquids, η is usually considered sufficiently close to zero to be ignored, especially given that these low-*Q* limits should only be treated as indicative for real materials.

Function	Low *Q*	Monatomic low *Q*	High *Q*	Units
*S*(*Q*)	\eta+1-{{\langle b_{\rm coh}^{2}\rangle} /{\langle b_{\rm coh}\rangle^{2}}}	η	1	Unitless
*F*^PDF^(*Q*)	0	0	0	Å^−1^
*F*_K_(*Q*)	\langle b_{\rm coh}\rangle^{2}\eta-\langle b_{\rm coh}^{2}\rangle	b_{\rm coh}^{2}(\eta-1)	0	Barn

**Table 3:** Average neutron scattering length constants for selected materials calculated using neutron scattering lengths and cross sections provided by Sears (1992 ▸) Note that 1 barn = 10^−24^ cm^2^ = 100 fm^2^.

Peterson *et al.* (2021) notation	Keen (2001 ▸) notation	MnO value (barn)	BaTiO_3_ value (barn)	SiO_2_ value (barn)
\langle b_{\rm tot}^{2}\rangle	\textstyle\sum^{n}_{i = 1}c_{i}\overline{b^{2}_{i}}	0.254	0.325	0.282
\langle b_{\rm coh}^{2}\rangle	\textstyle\sum^{n}_{i = 1}c_{i}\bar{b}^{2}_{i}	0.238	0.277	0.282
〈*b*_coh_〉^2^	{(}\textstyle\sum^{n}_{i = 1}c_{i}\bar{b}_{i}{)}^{2}	0.011	0.145	0.276

**Table 4:** List of reciprocal-space limits for selected materials with the assumption that η = 0 Although many materials have an *S*(*Q*) with a limiting value at low *Q* close to zero (*e.g.* SiO_2_), for materials containing elements with negative neutron coherent scattering lengths (*e.g.* MnO and BaTiO_3_) \lim_{Q\rightarrow 0}S(Q) is often far from zero.

Material	Function	Low *Q*	High *Q*
MnO	*S*(*Q*)	−21.1	1
	*F*^PDF^(*Q*)	0	0
	*F*_K_(*Q*)	−0.237	0
BaTiO_3_	*S*(*Q*)	−0.911	1
	*F*^PDF^(*Q*)	0	0
	*F*_K_(*Q*)	−0.277	0
SiO_2_	*S*(*Q*)	−0.022	1
	*F*^PDF^(*Q*)	0	0
	*F*_K_(*Q*)	−0.282	0
